# Data-driven health deficit assessment improves a frailty index’s prediction of current cognitive status and future conversion to dementia: results from ADNI

**DOI:** 10.1007/s11357-022-00669-2

**Published:** 2022-10-19

**Authors:** Andreas Engvig, Luigi A. Maglanoc, Nhat Trung Doan, Lars T. Westlye, Alzheimer’s Disease Neuroimaging Initiative

**Affiliations:** 1 NORMENT, Division of Mental Health and Addiction, Oslo University Hospital & Institute of Clinical Medicine, University of Oslo, Oslo, Norway; 2 Department of Nephrology, Oslo University Hospital, Oslo, Ullevål Norway; 3 University Center for Information Technology, University of Oslo, Oslo, Norway; 4 Department of Psychology, University of Oslo, Oslo, Norway

**Keywords:** Alzheimer’s disease, Dementia, Mild cognitive impairment, Frailty, Machine learning, Frailty index

## Abstract

Frailty is a dementia risk factor commonly measured by a frailty index (FI). The standard procedure for creating an FI requires manually selecting health deficit items and lacks criteria for selection optimization. We hypothesized that refining the item selection using data-driven assessment improves sensitivity to cognitive status and future dementia conversion, and compared the predictive value of three FIs: a standard 93-item FI was created after selecting health deficit items according to standard criteria (FI_s_) from the ADNI database. A refined FI (FI_r_) was calculated by using a subset of items, identified using factor analysis of mixed data (FAMD)-based cluster analysis. We developed both FIs for the ADNI1 cohort (*n* = 819). We also calculated another standard FI (FI_c_) developed by Canevelli and coworkers. Results were validated in an external sample by pooling ADNI2 and ADNI-GO cohorts (*n* = 815). Cluster analysis yielded two clusters of subjects, which significantly (p_FDR_ < .05) differed on 26 health items, which were used to compute FI_r_. The data-driven subset of items included in FI_r_ covered a range of systems and included well-known frailty components, e.g., gait alterations and low energy. In prediction analyses, FI_r_ outperformed FI_s_ and FI_c_ in terms of baseline cognition and future dementia conversion in the training and validation cohorts. In conclusion, the data show that data-driven health deficit assessment improves an FI's prediction of current cognitive status and future dementia, and suggest that the standard FI procedure needs to be refined when used for dementia risk assessment purposes.

**Supplementary Information:**

The online version contains supplementary material available at 10.1007/s11357-022-00669-2.

## Introduction

Frailty is an age-related state of multisystem physiological decline increasing the risk of adverse outcomes such as hospital complications and death [1–3]. The frailty index (FI) operationalizes frailty along a continuum based on the accumulation of health deficits model [4]. Evidence suggests that FIs may predict conversion to dementia [2, 5, 6] and points to FIs as useful measures for identifying subjects at high dementia risk. As frailty may be reversible [7], it is conceivable that frailty may serve as a target for dementia prevention. FIs are suitable for measuring frailty in this regard, as they are validated for longitudinal assessment [8] and show promise as outcome measures in clinical trials [9].

Standard FIs comprise a selection of several age-related health deficits which fit pre-defined criteria [10], and, when reflecting the accumulated burden of 30 to 40 health deficit variables, are robust for prediction of mortality [11]. In terms of dementia risk prediction, to the best of our knowledge, FI studies with external validation have so far been lacking. Traditionally, the deficit accumulation model has included more cognition-related measures when operationalizing frailty compared with its rival, the phenotype model [20]. Although frailty and dementia are inter-related, they are distinct concepts and by including dementia-related measures (e.g., certain activities of daily living, cognitive test results) into an FI, its use in the prediction of dementia may become circular. While Ward and coworkers found a significant association between FI and future dementia risk when adjusting for global cognition [12], results across studies show, however, that the association between FIs and future dementia risk weakens after removing deficits which might represent early core dementia symptoms [6, 12]. How to construct an optimal FI for dementia risk prediction purposes remains unknown. One caveat is that the standard procedure lacks criteria for discarding deficits with little explained variance, which may reduce FI performance [14].

In general, one approach to maximize explained variance is by employment of data-driven techniques which decompose and weigh correlated input variables. While the accuracy of a neural-network based machine learning approach outperformed an unweighted (i.e., standard) FI in the prediction of mortality [15], the application of machine learning weights to individual patients has been intangible. Using machine learning to guide selection of health deficit items at the development stage of an FI may represent a more feasible approach. The resulting FI may in turn be applied to any subject for which all or a subset of the relevant health deficit data exists. In general, discarding non-informative variables is an efficient step to remove noise and improve model performance. As an example, a data-driven refined FI based on a selection of 35-items identified using factor analysis among a larger set of items nearly outperformed a 139-item standard FI in terms of mortality prediction [16]. In contrast to factor analysis, principal component analysis (PCA) aims to maximize explained variance in variable set and may be used for weighting health deficits [17]. As most FIs include mixed data, factor analysis of mixed data (FAMD)––a combination of PCA for continuous variables and multiple correspondence analysis (MCA) for categorical––is needed [18, 19]. FAMD has thus far, however, not been applied to the field of frailty assessment.

**Fig. 1:**
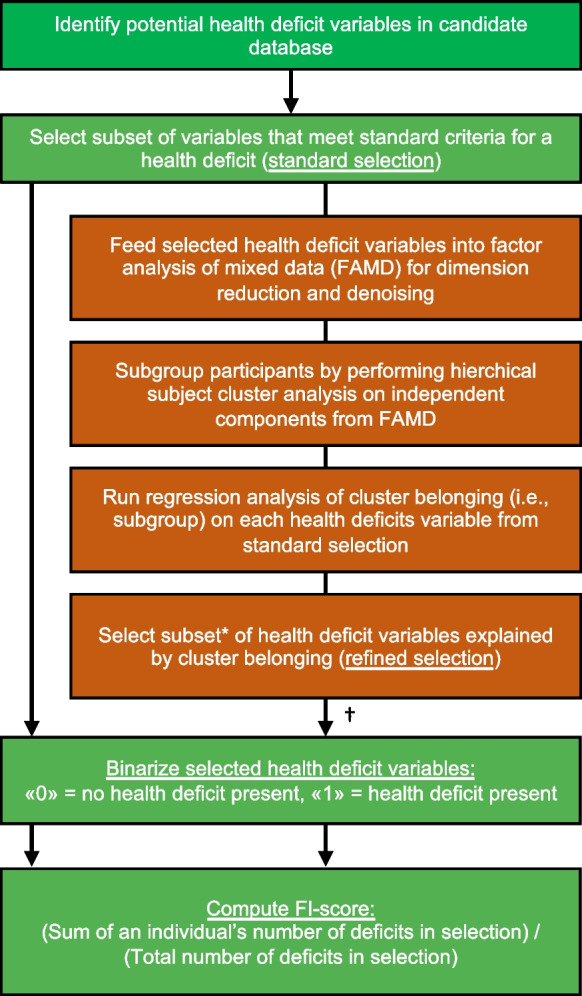
Data-driven supplement (red boxes) to the standard procedure (green boxes) for creating a frailty index (FI). *For the refined selection of health deficit variables, a false discovery rate (FDR)-adjusted p value < .05 from regression analyses of each variable on cluster belonging is used as selection threshold. †Assess face validity of refined selection against standard criteria and core frailty construct. FI_r_ (see “Methods” section) was developed using the data-driven supplement to the standard procedure as shown, whereas FI_s_ and FI_c_ were developed using standard procedure only

To this end, the purpose of the present study was to test adding FAMD-based cluster analysis to the standard FI procedure as a way to empirically guide health deficit selection (Fig. 1). Cluster analysis has previously been shown to identify subjects living with frailty in an unsupervised manner [20]. We hypothesized that data-driven health deficit selection would (1) improve the stability and replicability of an FI compared with standard procedure only, and (2) enhance its prediction of cognitive impairment, even when adjusting for cognitive and functional performance using a validated Clinical Dementia Rating (CDR) scale. To test our hypotheses, we created a data-driven FI (FI_r_) by applying FAMD-based clustering to a set of health variables selected according to standard criteria [10] from the ADNI database. The main objective was to develop and externally validate FI_r_ and compare it to two standard FIs using the ADNI1 (development) and ADNI2 and ADNI-GO (validation) cohorts against cognitive status and future dementia risk.

## Methods

### Data source

For model training, we used data from ADNI1, an observational prospective case–control cohort study (hereafter denoted “development sample”) of subjects between ages 55 to 90 living with normal cognition, mild cognitive impairment (MCI) or Alzheimer's disease dementia (AD). For external validation, we pooled participants from two other prospective cohorts, ADNIGO and ADNI2. All data are publicly available (https://ida.loni.usc.edu/)*.* The ADNI dataset used herein was downloaded on October 21st, 2021. ADNI was launched in 2003 as a public–private partnership, led by Principal Investigator Michael W. Weiner, MD. The primary goal of ADNI has been to test whether serial magnetic resonance imaging (MRI), positron emission tomography (PET), other biological markers, and clinical and neuropsychological assessment can be combined to measure the progression of MCI and AD. For up-to-date information, see www.adni-info.org.

#### Sample

Inclusion criteria for all participants included age 55 to 90 years; study partner to provide evaluation of function; speaks English; ability to undergo all testing, blood samples for genotyping and biomarkers, and neuroimaging procedures; completed six grades of education or work history; for women postmenopausal or surgically sterile, not depressed, and a modified Hachinski score less than five in order to rule out vascular dementia. Individuals with AD satisfied criteria for NINCDS/ADRDA for probable AD. Subjects enrolled as MCI had memory complaints verified by a study partner, Mini Mental Status Examination (MMSE) score of 24 to 30, Clinical Dementia Rating (CDR) score = 0.5 with sum of boxes (CDRSB) score of at least 0.5, and general cognition and functional performance sufficiently preserved such that a diagnosis of dementia could not be made. In later phases of ADNI, subjects with MCI were further sub-divided by their Wechsler Memory Scale Logical Memory II score into early (EMCI) and late (LMCI): in the present study, EMCI and LMCI-subjects were pooled and merely labeled MCI. Healthy controls (HC) had no memory complaints aside from those common to other normal subjects of that age range, MMSE score of 24 to 30, CDR = 0 (with CDRSB score = 0), and were deemed cognitively normal, based on an absence of significant impairment in cognitive functions or activities of daily living. Description of the enrollment is found online (http://www.adni-info.org/Scientists/doc/ADNI_GeneralProceduresManual.pdf).

### Statistical analyses

FI computation and statistical analyses were performed in R version 3.6.2. A github-repo is freely available online with the code used to generate the FIs and to perform the analyses detailed below (https://github.com/LAMaglan/ADNI-FI-clustering). An FI is based on a cumulative deficits model grading heterogeneity in health status on a continuous scale from 0 to 1, where greater scores indicate higher degree of frailty [10]. We selected candidate health deficits by assessing screening and baseline variables in the ADNI database. The selection process adhered to standard criteria described by Searle and coworker [10]. Specifically, we included variables reflecting health deficits, i.e., symptoms, clinical signs, diseases, laboratory abnormalities, or other measures associated with adverse health outcomes. Jointly, the deficits should cover many organ systems and consist of more than co-morbidities or function. The selected deficits should generally be considered age-related at the population level, but a variable representing a deficit does not, individually, need to be significantly related to age [11]. We selected health deficits which were present in at least 1% of the sample, but not more than 80%, and discarded variables which were missing in more than 5% of the patients. In accordance with previous research on frailty and cognitive outcomes by the developers of the FI method, we excluded certain health deficits related to the diagnosis of dementia [6, 21]. Specifically, we excluded neuropsychological test results, the remembering item in the Functional Activities Questionnaire (FAQ) and specific neuropsychiatric inventory (NPI) items such as disinhibition and delusions. A total of 93 health deficit variables fit the criteria and were used to compute a standard FI, denoted FI_s_. Next, we optimized the initial selection of health deficits using a data-driven refinement process (Fig. 1):

1. Factor analysis of mixed data (FAMD): we used FAMD (Pagès, 2004) in the R package *FactoMineR* [19] to reduce the 93 variables into principal components (PC). FAMD is a combination of principal component analysis (PCA) for continuous variables and multiple correspondence analysis (MCA) for categorical variables. All variables were normalized prior to the dimensionality reduction. Missing data for the continuous variables were imputed for dimensionality reduction using the k-Nearest Neighbour algorithm from the R package *bnstruct* [22]*.*
2. Subject cluster analysis: the number of PCs from FAMD explaining 80% cumulative variance was used as input for cluster analysis, which is in line with previous clinical studies employing FAMD-based clustering [23, 24]. Above this level, cumulative explained variance as function of PC number gradually plateaus (Supplementary Figure S1). For clustering, we used Hierarchical Clustering on Principal Components (HCPC) in the R-package *FactoMineR* [19]. HCPC entails agglomerative hierarchical clustering in the first step and k-means clustering to improve the initial clustering. Note that the hierarchical cluster analysis is performed on PCs following FAMD, and not directly on the original health deficit variables themselves. In addition to dimensionality reduction, this additional step is done to reduce noise in the data and generally yields a more stable cluster analysis [25]. We chose a two-cluster solution based on the higher relative loss of the sum of within-cluster inertia [19]. This entails that all participants are empirically divided into two cluster sub-groups.
3. Regression analysis for health deficit weighting: we used regression models to rank the relative importance or “weight” of the 93 health deficit variables. To this end, cluster sub-group belonging was used as independent binominal variable and each of the health deficit variables identified following the standard procedure as dependent variables. For binomial dependent variables, we used logistic regression models; for ordinal categorical variables (FAQ items), we used ordinal logistic regression; and for the continuous variables, we used linear regression.

To compute FI_r_, we included health deficit variables with a false discovery rate (FDR)-adjusted *p* < 0.05 from the regression analyses in step 3 only (i.e., the highest ranked items). We assessed face validity of the refined set of health deficits included in FI_r_ against standard criteria for an FI [10] and core components of the frailty construct [1].

In order to compare our FIs with one created by others, we further calculated a published 40-item FI (FI_c_) [26]. The items included in each FI is reported in Table 1. For all FIs, selected health deficit variables were binarized with a value of “1” given if a health deficit––or a marker thereof––was present, and “0” if absent. Continuous health deficit variables were dichotomized using established reference ranges, coding “1” as outside the normal range, “0” as within. For coding of continuous blood tests results, we used the ADNI laboratory reference ranges (including age and/or sex-specific cut-points). For activities of daily living variables, the deficit item was recoded in an ordinal manner [26]: for instance, the Finances item of the FAQ was coded as follows: “0” for independent/normal functioning, “0.25” for difficulty, and “0.5” for requires assistance, and “1” for dependent. All cut points used for dichotomization are provided in Supplementary Tables S1 and S2. Note that an FI score was only calculated for individuals who had less than 20% missing variables. The same selection of health deficits used to compute the FIs in the development sample (i.e., the ADNI1-cohort) were subsequently used to calculate corresponding FI-variables for the validation sample (i.e., pooled ADNI2 and ADNI-GO cohorts). The validation sample FIs were tested and displayed along with the results from the development sample for out-of-sample verification.

**Table 1:** Health deficit items included in FI_s_, FI_r_, and FI_c_

	Frailty Index (FI) variable		
Health deficit variables	FI_s_	FI_r_	FI_c_
On medication for arthritis	X		
On medication for elevated cholesterol	X		
On medication for hypertension	X		
On medication for hypothyroidism	X		
History of cardiovascular disease	X		X
History of endocrine-metabolic disease	X		X
History of head, eyes, ears, nose, and throat diseases	X		X
History of dermatologic-connective tissue disease	X		X
History of gastrointestinal disease	X		X
History of hematopoietic-lymphatic disease	X		X
History of hepatic disease	X		X
History of malignancies	X		X
History of musculoskeletal disease	X		X
History of neurological (non-AD) disease	X		X
History of psychiatric disease	X	X	X
History of renal-genitourinary disease	X		X
History of respiratory disease	X		X
History of other diseases	X		
History of major surgical procedures	X		
FAQ, writing checks, paying bills, or balancing checkbook	X	X	X
FAQ, assembling tax records, business affairs, or other papers	X	X	X
FAQ, heating water, making a cup of coffee	X	X	X
FAQ, traveling out of the neighborhood)	X	X	X
FAQ, preparing a balanced meal	X	X	X
FAQ, paying attention to and understanding a TV program, book, or magazine	X	X	X
FAQ, playing a game of skill such as bridge or chess	X	X	X
FAQ, shopping alone for clothes, household	X	X	X
NPI, agitation	X	X	X
NPI, anxiety	X	X	X
NPI, depression	X	X	
NPI, irritability	X	X	X
NPI, sleep disturbance	X	X	
NPI, apathy	X	X	X
NPI, aberrant motor behavior	X	X	X
NPI, change in appetite and eating	X	X	
Auditory impairment on physical exam	X		
Cranial nerve abnormality on physical exam	X		
Abnormal finger to nose test	X		X
Abnormal heel-knee test	X		
Abnormal gait on physical exam	X	X	X
Motor strength deficit on physical exam	X		
Abnormal plantar reflex on physical exam	X		
Abnormal tendon reflex on physical exam	X		
Sensory nerve abnormality on physical exam	X		
Tremor on physical exam	X		X
Vision impairment on physical exam	X		
Symptoms from abdomen	X		
Self-reported ankle swelling	X		
Self-reported shortness of breath	X		X
Self-reported chest pain	X		
Self-reported constipation	X		X
Self-reported cough	X		
Self-reported depressed mood	X	X	
Self-reported dizziness	X		X
Self-reported drowsiness	X	X	X
Self-reported dry mouth	X		
Self-reported low energy	X	X	X
Self-reported recent fall	X		X
Self-reported insomnia	X		X
Self-reported life satisfaction	X		
Self-reported muscle pain	X		X
Self-reported palpitations	X		
Self-reported urinary discomfort	X		X
Self-reported urinary frequency	X		
Self-reported vision disturbance	X		
Blood albumin (g/dL)	X		
Blood alkaline phosphatase (ALP; U/L)	X		
Blood alanine aminotransferase (ALT; U/L)	X		
Blood aspartate aminotransferase (AST; U/L)	X		
Blood vitamin B12 (pg/mL)	X		
Blood total bilirubin (mg/dL)	X		
Blood calcium (mg/dL)	X		
Blood cholesterol (mg/dL)	X		
Blood creatinine (mg/dL)	X		
Blood glucose (mg/dL)	X		
Blood hematocrit (%)	X		
Blood hemoglobin (g/dL)	X		
Blood mean corpuscular hemoglobin (MCH; pg)	X		
Blood mean corpuscular volume (MCV; fL)	X		
Blood neutrophil count (10^3^/μl)	X	X	
Blood total protein (g/dL)	X		
Blood red blood cell count (RBC; 10^6^/μl)	X	X	
Blood triglycerides (mg/dL)	X		
Blood urea nitrogen (BUN, mg/dL)	X		
Blood uric acid (mg/dL)	X		
Body mass index (BMI)	X		
Diastolic blood pressure (mmHg)	X		X
Heart rate (count)	X		
Mean arterial pressure (mmHg)	X		
Pulse pressure (mmHg)	X	X	
Systolic blood pressure (mmHg)	X		X
Number of medications (polypharmacy)	X	X	
Elevated geriatric depression scale (GDS) score	X	X	

*X* denotes that the variable is included in the respective frailty index (FI). *FI*_*s*_ = a 93-item frailty index created according to standard procedure. *FI*_*r*_ = a 26-item frailty index created according to a refined, data-driven procedure. *FI*_*c*_ = a 40-item FI created according to standard procedure by Canevelli, et al. [26]. *FAQ* = Functional Assessment Questionnaire. *NPI* = Neuropsychiatric Inventory

#### Diagnostic prediction performance

To assess diagnostic performance of the two standard FIs (FI_s_ and FI_c_) versus FI_r_, we performed three sets of machine-learning based binary classification on the diagnostic groups (HC vs AD, MCI vs AD, and HC vs MCI) using linear discriminant analysis from the R packages *discrim* [27] and *tidymodels* [28]. In the main analyses, we ran tenfold internal cross-validation on the ADNI1 data (i.e., whereby 9 of the folds predict the remaining fold iteratively), repeated 100 times on randomly partitioned data. To assess reproducibility, we built our models based on the development cohort (ADNI1) which were then used to classify the diagnostic groups in the validation cohort (ADNI2 and ADNI-GO). To obtain an estimate of standard deviation for each of the binary classification analyses of the validation-sample, we performed a pseudo-tenfold cross-validation with 100 repetitions, where first the validation-sample was split into 10 folds, where each of the folds was predicted with the same machine learning model (built on the whole development-sample, i.e., ADNI1 dataset) for each of the respective binary classification tasks. We computed area under the curve (AUC) as our main measure of model performance, but also report sensitivity, specificity, negative predictive value (NPV), positive predictive value (PPV), and the F1-score. Due to differences in diagnostic group size (i.e., class imbalance), we conducted sensitivity analyses to account for imbalanced sampling in the development-sample. Here, for every binary classification task, we undersampled the larger diagnostic group to match the lower diagnostic group. Then, as in the main analyses in the development sample, we ran tenfold cross-validation with 100 repetitions.

Finally, we examined whether the discriminative ability of the three FI-variables changed according to different age and sex strata by dividing the development sample into young-old and old-old (± 75 years), and male and female. Here, we performed binary classification on the four resulting sub-groups for HC versus AD classification only to constrain the number of analyses run. We ran paired *t* tests to statistically compare the model performance of the two classifiers (either FI_s_ or FI_c_, versus FI_r_) in the main analyses, as well as for the sensitivity analyses with undersampling.

#### Prognostic performance

We evaluated how well the FI-variables predicted future dementia risk by survival analysis of subjects living with MCI at baseline. Analyses were performed using the *survival* package [29], if not otherwise stated. We assessed time from baseline examination to the date of registered AD conversion. Participants who had not progressed at their last recorded visit were right-censored. We did not account for a competing mortality risk. Dementia-free survival in MCI participants was first assessed by different sample levels of frailty using FI-quartiles without adjusting for covariates by the Kaplan–Meier estimator. To test and compare the predictive performance of the three continuous FI-variables over time we also estimated time-dependent areas under receiver operating characteristic curves (AUC(t)) and their 95% confidence intervals and bands by means of the R package ‘*timeROC*’, using the iid-representation of the AUC estimator for inference [30].

Next, we tested associations between dementia-free survival and the three FIs as continuous variables by fitting multivariate Cox proportional hazards models, taking into account age, sex, education as well as baseline cognitive and functional performance. Here, FI-scores were multiplied by 100 in order to facilitate meaningful interpretation of the associated hazard ratios (HR). In the first model set, we included either one of the continuous FI variables, covarying for age, sex, and education. In the second model set, we assessed relationships between the FI scores and dementia risk when accounting for baseline cognitive and functional performance. To this end, we fitted Cox proportional hazards models with the Clinical Dementia Rating Sum of Boxes (CDRSB) score for each participant as an additional covariate. For the development sample, individual, and global Schoenfeld scaled residuals tests were all non-significant, suggesting proportionality of hazards. In the validation sample, Schoenfeld scaled residuals tests indicated violation of the proportionality of hazards assumption for the age covariate (*p* < 0.05). Thus, for the multivariate validation models, we estimated average hazard ratios [31] using Prentice weights with censoring correction and robust variance estimation as implemented in the *coxphw* package.

To examine predictive validity, we tested associations between the FI-variables and mortality risk—the endpoint for which the FI-approach was originally developed [4, 10]. Due to a relatively low mortality rate, particularly in the validation cohort (*n*_deaths_ = 15 AD, 10 HC and 20 MCI, respectively), subjects across diagnostic groups were pooled and the analyses were considered explorative. We hypothesized that FI_r_ would be associated with mortality risk comparable to published literature, also when adjusting for confounders (age, sex, education, cognitive performance). Here, MMSE was used to account for variation in baseline cognition instead of CDRSB as the latter equals 0 in HC subjects. In the adjusted analyses, we assumed non-proportional hazards and estimated average hazard ratios as described above since Kaplan–Meier plots showed pronounced crossing of the survival curves for FI_s_ and FI_c_ (Supplementary Figure S4).

## Results

Table 2 summarizes baseline characteristics for the development and validation samples. The validation sample was younger, more highly educated, and included fewer patients living with dementia at baseline compared with the development sample.

**Table 2:** Baseline characteristics of development and validation samples

Sample	Development (ADNI1, *n* = 819)	Validation (ADNI2 + GO, *n* = 815)	*p*
Baseline diagnosis, *n* (%)			< 0.001
AD	193 (23.6%)	151 (18.5%)	
HC	229 (28.0%)	190 (23.3%)	
MCI	397 (48.5%)	474 (58.2%)	
Age in years, median [IQR]	75.5 [71.2;80.0]	72.6 [67.6;77.7]	< 0.001
Sex, *n* (%)			0.075
Female	342 (41.8%)	377 (46.3%)	
Male	477 (58.2%)	438 (53.7%)	
Education in years, median [IQR]	16.0 [13.0;18.0]	16.0 [14.0;18.0]	< 0.001
MMSE, median [IQR]	27.0 [25.0;29.0]	28.0 [26.0;29.0]	< 0.001
CDRSB, median [IQR]	1.5 [0.0;3.0]	1.0 [0.5;2.5]	0.400
FI_s_			
Median [IQR]	0.168 [0.125;0.220]	0.174 [0.133;0.228]	0.019
Mean (SD)	0.176 (0.070)	0.183 (0.067)	
99th percentile	0.358	0.367	
FI_r_			
Median [IQR]	0.154 [0.077;0.269]	0.154 [0.096;0.269]	0.125
Mean (SD)	0.187 (0.138)	0.192 (0.131)	
99th percentile	0.615	0.594	
FI_c_			
Median [IQR]	0.200 [0.131;0.269]	0.206 [0.150;0.275]	0.349
Mean (SD)	0.211 (0.100)	0.214 (0.097)	
99th percentile	0.469	0.468	

*AD* = Alzheimer’s disease dementia. *FI*_*s*_ = a 93-item frailty index (FI) created according to standard procedure by the authors. *FI*_*r*_ = a 26-item FI created by adding a data-driven supplement to the standard procedure. *FI*_*c*_ = a 40-item FI created according to standard procedure by Canevelli, et al. [26]. *HC* = cognitively normal control. *IQR* = interquartile range. *MCI* = mild cognitive impairment. *SD* = standard deviation. Sample group differences for continuous and categorical variables were assessed by Kruskal–Wallis and Pearson’s chi-squared tests, respectively

### FAMD-based subject cluster analysis

**Fig. 2:**
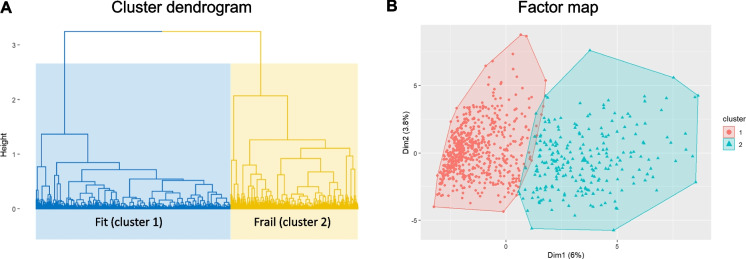
**A** Dendrogram showing the hierarchical structure of the subject clustering solution. The blue part shows the first and largest cluster which we coined “fit” due to significantly lower frailty scores in this subgroup (c.fr. Table 3) compared with the second, smaller cluster (yellow), coined “frail”. **B** Scatterplot showing subjects and their cluster belonging based on the two first principal components (PC, “Dim1”, “Dim2”) from factor analysis of mixed data (FAMD). The percentages denote explained variance of each PC

We performed FAMD on the 93 health deficit variables identified by the standard procedure which yielded 93 PCs. A plot of the cumulative explained variance of all PCs, and a scree plot of the first 10 are shown in Supplementary Figure S1. The contribution of each continuous and categorical health deficit variable to the first 5 PCs are shown in Supplementary Figures S2 and S3, respectively. FAQ-items contributed most to the 1st PC. Number of prescription drugs contributed the most to the 2nd. The first 60 PCs, explaining 80% of cumulative variance, were fed into cluster analysis. A cluster dendrogram showing the empirical division of individual subjects into our chosen two-cluster solution is shown in Fig. 2. Baseline characteristics of the individuals in each cluster are shown in Table 3. Median degree of frailty, as measured by two standard FIs (FI_s_, FI_c_), was significantly greater in the smaller cluster (cluster 2, coined “frail”), comprising 230 subjects (149 AD, 81 MCI). The largest cluster, cluster 1 (coined “fit”), consisted of 589 subjects (229 HC, 316 MCI, and 44 AD). The participants in the two clusters were of comparable age, and had similar sex distributions. In cluster 2, more subjects were living with polypharmacy, more reported low levels of energy, and had more symptoms of depression compared with subjects in cluster 1.

**Table 3:** Baseline characteristics of subject cluster sub-groups formed by FAMD-based cluster analysis

Cluster	Cluster 1 **“**Fit**”**	Cluster 2 **“**Frail**”**	*p* value
	(*N* = 589)	(*N* = 230)	
Diagnosis			< 0.001
-AD	44 (7.5%)	149 (64.8%)	
-HC	229 (38.9%)	0 (0.0%)	
-MCI	316 (53.7%)	81 (35.2%)	
Age	75.2 [71.3;79.7]	76.40 [70.70;81.80]	0.299
Sex			0.943
-Female	245 (41.6%)	97 (42.2%)	
-Male	344 (58.4%)	133 (57.8%)	
Education, years	16.0 [14.0;18.0]	16.0 [12.0;17.0]	0.001
CDRSB	0.50 [0.00;1.50]	3.50 [2.50;5.00]	< 0.001
FI_s_	0.15 [0.11;0.19]	0.22 [0.18;0.27]	< 0.001
FI_c_	0.17 [0.12;0.23]	0.30 [0.23;0.37]	< 0.001
Number of prescription drugs]	5.0 [3.0;7.0]	6.0 [4.0;8.0]	< 0.001
Self reported low energy			< 0.001
-Absent	504 (85.6%)	154 (67.0%)	
-Present	85 (14.4%)	76 (33.0%)	
NPI, depressive symptoms			< 0.001
-No	524 (89.1%)	140 (60.9%)	
-Yes	64 (10.9%)	90 (39.1%)	

*AD* = Alzheimer’s disease dementia. *CDRSB* = Clinical Dementia Rating Sum of Boxes. *FI*_*s*_ = a 93-item frailty index (FI) created according to standard procedure by the authors. *FI*_*c*_ = a 40-item FI created according to standard procedure by Canevelli, et al. [26]. *HC* = healthy cognitively normal control. *MCI* = mild cognitive impairment. Continuous variables are reported as median [interquartile range]. Sample group differences for continuous and categorical variables by Kruskal–Wallis and Pearson’s chi-squared tests, respectively

Table 4 shows the empirical ranking of health deficit items, including the 26 health deficit variables correlating significantly (p_FDR_ < 0.05) with cluster belonging, which were used to compute FI_r_. Sixteen out the 26 health deficits used to compute FI_r_ were related to activities of daily living (FAQ) and neuropsychiatric symptoms (NPI) reported by next of kin. Three items were based on self-report (feeling depressed, low energy, drowsy). One item was based on depressive symptoms as rated on the geriatric depression scale (GDS), one was having abnormal gait on physical exam, one was number of prescription drugs, two were blood test results (neutrophil count, red blood cell count), the last item was pulse pressure. Except having an item count less than 30, FI_r_ was deemed in accordance with standard criteria for an FI [10].

**Table 4:** Ranking of health deficit items

Rank	Health deficit variable	Adjusted *p* value
1	FAQ, writing checks, paying bills, or balancing checkbook	< 0.001
2	FAQ, assembling tax records, business affairs, or other papers	< 0.001
3	FAQ, shopping alone for clothes, household	< 0.001
4	FAQ, traveling out of the neighborhood)	< 0.001
5	FAQ, preparing a balanced meal	< 0.001
6	FAQ, playing a game of skill such as bridge or chess	< 0.001
7	FAQ, paying attention to and understanding a TV program, book, or magazine	< 0.001
8	FAQ, heating water, making a cup of coffee	< 0.001
9	NPI, anxiety	< 0.001
10	NPI, apathy	< 0.001
11	NPI, agitation	< 0.001
12	NPI, depression	< 0.001
13	NPI, irritability	< 0.001
14	NPI, change in appetite and eating	< 0.001
15	NPI, sleep disturbance	< 0.001
16	Self-reported depressed mood	< 0.001
17	NPI, aberrant motor behavior	< 0.001
18	History of psychiatric disease	< 0.001
19	Self-reported low energy	< 0.001
20	Self-reported drowsiness	< 0.001
21	Geriatric Depression Scale (GDS) score	< 0.001
22	Abnormal gait on physical exam	< 0.001
23	Number of medications (polypharmacy)	< 0.001
24	Blood neutrophil count (10^3^/μl)	< 0.001
25	Blood red blood cell count (RBC; 10^6^/μl)	0.01
26	Pulse pressure (mmHg)	0.03
27	Blood glucose (mg/dL)	0.07
28	Self-reported dizziness	0.07
29	History of endocrine-metabolic disease	0.07
30	Heart rate (count)	0.07
31	Blood hematocrit (%)	0.07
32	Tremor on physical exam	0.12
33	Body mass index (BMI)	0.12
34	Blood mean corpuscular hemoglobin (MCH; pg)	0.16
35	Blood albumin (g/dL)	0.16
36	Blood mean corpuscular volume (MCV; fL)	0.20
37	Blood hemoglobin (g/dL)	0.20
38	Systolic blood pressure (mmHg)	0.24
39	Vision impairment on physical exam	0.28
40	Self-reported muscle pain	0.29
41	Blood alanine aminotransferase (ALT; U/L)	0.29
42	Blood alkaline phosphatase (ALP; U/L)	0.30
43	Blood creatinine (mg/dL)	0.30
44	Cranial nerve abnormality on physical exam	0.30
45	Blood cholesterol (mg/dL)	0.31
46	On medication for elevated cholesterol	0.31
47	On medication for arthritis	0.34
48	Diastolic blood pressure (mmHg)	0.42
49	Self-reported chest pain	0.49
50	On medication for hypertension	0.49
51	Self-reported dry mouth	0.50
52	Blood urea nitrogen (BUN, mg/dL)	0.52
53	Abnormal plantar reflex on physical exam	0.52
54	Mean arterial pressure (mmHg)	0.52
55	Blood triglycerides (mg/dL)	0.52
56	Self-reported recent fall	0.53
57	Auditory impairment on physical exam	0.53
58	History of musculoskeletal disease	0.55
59	History of hepatic disease	0.60
60	On medication for hypothyroidism	0.61
61	Self-reported cough	0.61
62	Abnormal finger to nose test	0.61
63	Self-reported constipation	0.62
64	Blood uric acid (mg/dL)	0.62
65	Self-reported palpitations	0.62
66	Abnormal tendon reflex on physical exam	0.68
67	History of gastrointestinal disease	0.71
68	Abnormal heel-knee test	0.72
69	Motor strength deficit on physical exam	0.72
70	Symptoms from abdomen	0.72
71	Sensory nerve abnormality on physical exam	0.72
72	Self-reported vision disturbance	0.72
73	Self-reported life satisfaction	0.72
74	History of head, eyes, ears, nose, and throat diseases	0.72
75	Self-reported urinary frequency	0.72
76	Self-reported shortness of breath	0.74
77	Blood calcium (mg/dL)	0.77
78	History of dermatologic-connective tissue disease	0.77
79	History of cardiovascular disease	0.77
80	Self-reported ankle swelling	0.77
81	Blood total protein (g/dL)	0.77
82	History of neurological (non-AD) disease	0.80
83	Blood total bilirubin (mg/dL)	0.84
84	Blood vitamin B12 (pg/mL)	0.89
85	History of respiratory disease	0.89
86	History of renal-genitourinary disease	0.89
87	History of other diseases	0.89
88	History of hematopoietic-lymphatic disease	0.89
89	History of major surgical procedures	0.97
90	Blood aspartate aminotransferase (AST; U/L)	0.97
91	History of malignancies	0.97
92	Self-reported urinary discomfort	0.97
93	Self-reported insomnia	0.99

The right column shows false discovery rate (FDR)-adjusted *p* values from regression analyses examining the association between 93 health deficits identified according to a set of standard criteria given by Searle, et al. [10], and subject clusters resulting from factor analysis of mixed data (FAMD)-based clustering analysis. Deficits were ranked according to the *p* values and a horizontal line below item 26 marks the 0.05 *p* value threshold. The top 26 items were used to compute FI_r_. *Abbreviations*: *FAQ* = Functional Assessment Questionnaire. *NPI* = Neuropsychiatric Inventory

### Frailty index characteristics

**Fig. 3:**
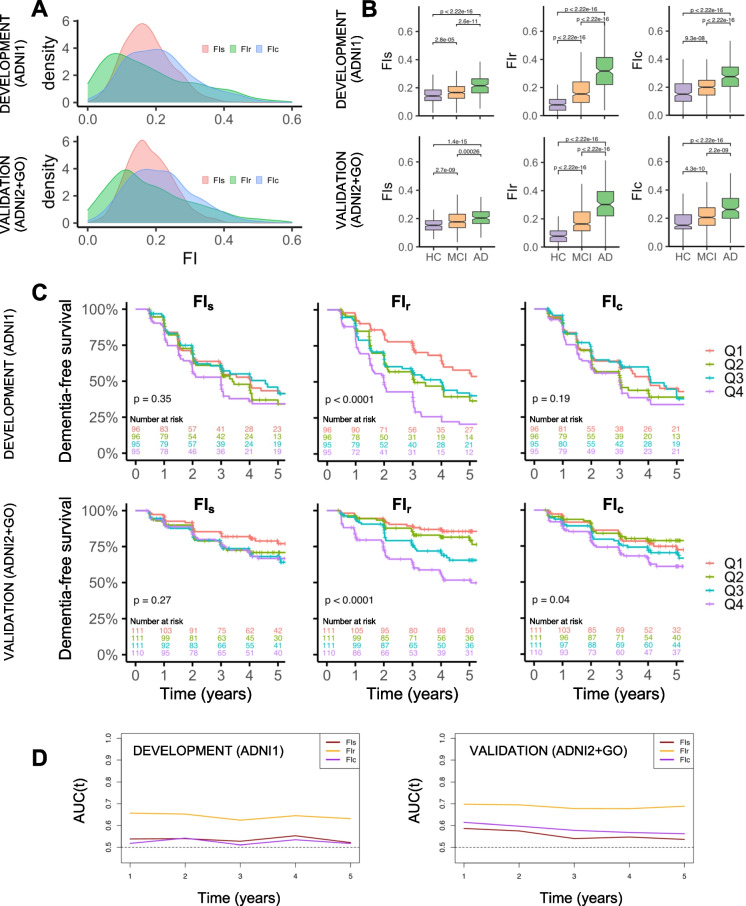
**A** Density plots showing three frailty index (FI) distributions for ADNI1 (development) and ADNI2/GO (validation) cohorts. FI_s_ = a 93-item FI created according to standard procedure by the authors. FI_r_ = a 26-item FI created by adding a data-driven supplement to the standard procedure. FI_c_ = a 40-item FI created according to standard procedure by Canevelli, et al. [26]. **B** Boxplots illustrating central tendency and variability of the three different FI-variables for cognitively normal (healthy) controls (HC), and subjects living with mild cognitive impairment (MCI) or Alzheimer’s disease dementia (AD). *P* values are from Wilcoxon rank sum tests comparing diagnostic group differences in FI-scores. **C** Kaplan–Meier survival curves for sample quartiles calculated for each FI. The survival probabilities indicate the probability of remaining stable MCI at time of follow-up, and vertical lines through each line indicate censoring. **D** Estimated mean AUC(t) for prediction of AD conversion in subjects with MCI at baseline plotted over 5 years of follow-up for the three rival continuous FI variables in development and validation cohorts

Density plots for all three FI-variables for the development and validation samples are shown in Fig. 3A, whereas central tendency, variability and 99th percentiles are quantified in Table 2. FI_r_ displayed a pattern of greater right-skew, variability and a higher upper FI-limit compared with standard FIs (FI_s_, FI_c_). As expected, median FI-scores increased with greater degree of cognitive impairment for all FIs (Fig. 3B). As shown in Supplementary Figure S5, the relationship with age appeared weaker for FI_r_ compared with standard FIs.

### Diagnostic performance

Table 5 shows the results from pairwise diagnostic group classifications for the development and validation samples. For classification of HC versus AD, FI_r_ showed excellent performance in both development and validation samples (AUC 0.95 and 0.93, respectively) with relatively balanced sensitivity and specificity. For all other group classifications, poorly balanced sensitivity and specificity were seen, reflected by the imbalanced diagnostic group sizes. Class imbalance correction by undersampling led to more balanced sensitivity and specificity metrics, while overall model performance remained largely unchanged (Supplementary Table S3). For HC versus MCI, both FI_s_ and FI_c_ showed poor classification performance in both samples, whereas FI_r_ showed acceptable performance. For all comparisons, including results obtained by adjusting for class imbalance, classification performance was greater for FI_r_ compared with both FI_s_ and FI_c_ (*p* < 0.001*)*. Assessment of discriminative ability across age and sex-strata (Supplementary Table S4) suggested poorer performance for FI_s_ in younger old (below 75 years) compared with old (75 +) age groups, particularly for males. A similarly tendency was seen for FI_c_, whereas overall performance for FI_r_ remained excellent across age and sex-strata. For females aged 75 and older, FI_r_ AUC for HC versus AD classification was outstanding (0.97, 95% CI 0.95 to 0.99).

**Table 5:** Pairwise group classification

Classification/performance measure	Sample					
	Development (ADNI1)			Validation (ADNI2 + GO)		
	FI-variable					
*HC vs. AD*	FI_s_	FI_r_	FI_c_	FI_s_	FI_r_	FI_c_
Area under the curve	0.75 (0.00)	0.95 (0.00)	0.81 (0.00)	0.75 (0.00)	0.93 (0.00)	0.80 (0.00)
Sensitivity	0.81 (0.00)	0.94 (0.00)	0.83 (0.00)	0.82 (0.00)	0.95 (0.00)	0.82 (0.00)
Specificity	0.56 (0.01)	0.77 (0.00)	0.64 (0.00)	0.53 (0.00)	0.76 (0.00)	0.58 (0.00)
PPV	0.69 (0.00)	0.83 (0.00)	0.74 (0.00)	0.69 (0.00)	0.83 (0.00)	0.71 (0.00)
NPV	0.71 (0.01)	0.92 (0.00)	0.77 (0.01)	0.70 (0.00)	0.92 (0.00)	0.72 (0.00)
F1-score	0.74 (0.00)	0.88 (0.00)	0.78 (0.00)	0.75 (0.00)	0.89 (0.00)	0.76 (0.00)
*MCI vs. AD*	FI_s_	FI_r_	FI_c_	FI_s_	FI_r_	FI_c_
Area under the curve	0.67 (0.00)	0.81 (0.00)	0.71 (0.00)	0.60 (0.00)	0.76 (0.00)	0.66 (0.00)
Sensitivity	0.97 (0.00)	0.89 (0.00)	0.92 (0.00)	0.95 (0.00)	0.89 (0.00)	0.91 (0.00)
Specificity	0.11 (0.01)	0.47 (0.00)	0.24 (0.00)	0.08 (0.00)	0.42 (0.00)	0.23 (0.00)
PPV	0.69 (0.00)	0.78 (0.00)	0.71 (0.00)	0.76 (0.00)	0.83 (0.00)	0.79 (0.00)
NPV	0.66 (0.05)	0.69 (0.01)	0.61 (0.02)	0.32 (0.00)	0.55 (0.00)	0.44 (0.00)
F1-score	0.81 (0.00)	0.83 (0.00)	0.80 (0.00)	0.85 (0.00)	0.86 (0.00)	0.84 (0.00)
*HC vs. MCI*	FI_s_	FI_r_	FI_c_	FI_s_	FI_r_	FI_c_
Area under the curve	0.60 (0.00)	0.78 (0.00)	0.63 (0.00)	0.65 (0.00)	0.78 (0.00)	0.65 (0.00)
Sensitivity	0.00 (0.00)	0.41 (0.00)	0.06 (0.01)	0.01 (0.00)	0.31 (0.00)	0.07 (0.01)
Specificity	1.00 (0.00)	0.89 (0.00	0.97 (0.00)	1.00 (0.00)	0.92 (0.00)	0.97 (0.00)
PPV	0.15 (0.03)^§^	0.68 (0.01)	0.57 (0.06)	1.00 (0.00)	0.60 (0.00)	0.45 (0.00)
NPV	0.63 (0.00)	0.73 (0.00)	0.64 (0.00)	0.71 (0.00)	0.77 (0.00)	0.72 (0.00)
F1-score	0.15 (0.03)^§^	0.51 (0.00)	0.18 (0.00)^§^	0.01 (0.00)	0.41 (0.01)	0.12 (0.05)

*AD* = Alzheimer’s disease dementia, *AUC* = area under the curve. *FI*_*s*_ = a 93-item frailty index (FI) created according to standard procedure by the authors. *FI*_*r*_ = a 26-item FI created by adding a data-driven supplement to the standard procedure. *FI*_*c*_ = a 40-item FI created according to standard procedure by Canevelli, et al. [26]. *HC* = healthy cognitively normal control. *MCI* = mild cognitive impairment. *NPV* = negative predictive value. *PPV* = positive predictive value

^§^Error margin is reported as standard error

### Prognostic performance

In the development sample, 382 out of 397 subjects with MCI at baseline had diagnostic follow-up data, with a median follow-up time of 744 days (interquartile range, 410 to 1461); 205 (54%) converted to dementia. In the validation sample, 443 out of 474 subjects with MCI at baseline had diagnostic follow-up data, with a median follow-up time of 1451 days (interquartile range, 731 to 2195); 111 (25%) converted to dementia.

Figure 3D shows Kaplan–Meier survival curves illustrating the probability of remaining stable MCI (dementia-free) at time of follow-up for different FI-quartiles calculated for each sample. The FI-quartile curves differed significantly in terms of future dementia risk across samples for FI_r_ only. Estimates of AUC(t) for prediction of AD conversion in subjects with MCI at baseline over 5-year follow-up for the three continuous FI variables are shown in Fig. 4E. AUC(t) 95% confidence bands (Supplementary Figure S6) for FI_s_ and FI_c_ both intersected the 0.5-line, suggesting poor to chance level discrimination. Average AUC(t) for FI_r_ ranged from 0.62–0.70 over time suggesting poor to acceptable performance in prediction of future dementia risk, outperforming FI_c_ and FI_s_ from year 2 in both samples (Supplementary Figure S7). In the multivariate survival analyses (Table 6), only FI_r_ associated with future dementia risk across samples and in a model adjusting for cognitive and functional baseline performance (as scored by CDRSB).

**Table 6:** Associations between three different FI-variables and conversion to dementia within follow-up for subjects with MCI at baseline

Sample/FI-variable*	Model 1^a^		Model 2^b^	
*Development* *(ADNI1)*	HR (95% CI)	*p* value	HR (95% CI)	*p* value
FI_s_	1.02 (1.00–1.04)	0.04	1.00 (0.98–1.03)	0.68
FI_r_	1.03 (1.02–1.05)	< 0.001	1.02 (1.01–1.03)	< 0.001
FI_c_	1.01 (1.00–1.03)	0.06	1.00 (0.99–1.02)	0.72
*Validation* *(ADNI2/GO)*^c^	AHR (95% CI)	*p* value	AHR (95% CI)	*p* value
FI_s_	1.02 (0.99–1.05)	0.19	0.99 (0.96–1.02)	0.40
FI_r_	1.04 (1.03–1.06)	< 0.001	1.02 (1.00–1.04)	0.02
FI_c_	1.02 (1.00–1.05)	0.04	1.00 (0.97–1.02)	0.80

*AHR* = average hazard ratio. *HR* = hazard ratio. *CI* = confidence interval. *FI*_*s*_ = a 93-item frailty index (FI) created according to standard procedure by the authors. *FI*_*r*_ = a 26-item FI created by adding a data-driven supplement to the standard procedure. *FI*_*c*_ = a 40-item FI created according to standard procedure by Canevelli, et al. [26]

^*^All FI-variables were multiplied by 100 before entered into the models

^a^Model 1 included age, sex, and education as covariates

^b^Model 2 included age, sex, education, and Clinical Dementia Rating scale, Sum of Boxes (CDRSB)

^c^Due to non-proportionality of hazards for age in model 1 and 2 of the validation cohort, we estimated average hazard ratios (AHRs) instead of HRs using Prentice weights with censoring correction and robust variance estimation

In exploratory analyses, we found a crude mortality hazard ratio for FI_r_ of 1.04 (1.02 to 1.05) which is in accordance with published estimates from FI meta-analysis (HR 1.04, 95% CI 1.03 to 1.04) [32]. Kaplan–Meier survival curves and associations with mortality risk for all three FI-variables (FI_s_, FI_r_, FI_c_) are shown in Supplementary Figure S4 and Supplementary Table S5. In the Development sample, only FI_r_ associated with mortality risk (average hazard ratio 1.02, 95% CI 1.01 to 1.04) in the fully adjusted model.

## Discussion

Several studies show promise for FIs in dementia risk prediction [2, 5, 6, 12], but the results have been variable and validation studies are lacking. We tested whether adding a data-driven health deficit selection step to the standard FI procedure improves prediction of current cognitive status and future conversion to dementia. The data-driven optimization procedure may help researchers and clinicians streamline FI development and improve early detection of MCI and AD and identify those at highest risk for clinical trial enrolment.

Our results show that the data-driven FI_r_ outperforms standard FIs in terms of diagnostic and prognostic performance, both in development and validation samples. As an example of diagnostic performance, Canevelli and coworkers reported an AUC of 0.67 to 0.75 for a standard FI in discriminating participants with and without dementia [26]. Classification performance for FI_r_ in discriminating subjects with normal cognition and dementia was 0.95 to 0.96. While FI_r_ diagnostic performance remained excellent (mean AUCs from 94 to 97) across age and sex-strata, overall performance dropped in younger strata for standard FIs (FI_s_, FI_c_). As the FAMD-based clustering approach employed here aims to maximize explained variance of included health deficits, overlap with confounding entities is likely diminished.

In terms of prognostic performance, only FI_r_ associated with future dementia conversion when adjusting for cognitive status. Some studies of frailty and future dementia risk have not adjusted for baseline cognitive status [2, 6], others have shown conflicting results [5, 12, 33]. The present study is the first to include out-of-sample verification. Our data show that two FIs created according to standard procedure had little added value in prediction of dementia when adjusting for cognitive performance, even when health deficits strongly related to dementia (such as certain ADL-items and cognitive tests) were excluded. The results show that dementia prediction ability of the accumulation of deficits model is variable and depends on the way an FI is constructed. Our proposed data-driven assessment of health deficits revealed items particularly sensitive to AD-related cognitive status and conversion to dementia. We argue that the standard procedure for creating an FI published in 2008 [10] could benefit from revision and that health deficit selection may be streamlined by FAMD-based subject clustering.

### Characteristics of health deficits identified by cluster analysis

In line with previous studies, higher FI-scores were associated with greater degree of cognitive impairment [5, 33, 34]. FAQ items were amongst the top health deficit variables that differed most between cluster subgroups, including independence in assembling tax records, business affairs, or other papers, and writing checks, paying bills, or balancing checkbooks. Previous studies confirm that FAQ is sensitive to early cognitive decline [35, 36]. Polypharmacy (more than five different prescription medications daily) was also among the highest ranked health deficit variables following cluster analysis, a feature of frailty previously associated with dementia risk [37]. Cardiovascular disease and frailty are closely linked and may share similar causal mechanisms [38]. As such, most FIs to date consist of one or more items involving the cardiovascular system, including a history of ischemic heart disease, stroke, or heart failure [e.g., 2, 5, 16, 21, 26, 34]. Some also include biomarkers thereof, such as blood pressure [21, 26]. The question here seems not to be *whether* cardiovascular health deficits should be included in an FI, but rather *which* one to choose in order to maximize FI performance. While the standard procedure for creating an FI gives little advice on *optimal* item selection [10], the present data-driven approach ruled out all cardiovascular items except pulse pressure (PP). Often used as a surrogate of arterial stiffness, increased PP is a feature of aging that has been associated with blood–brain barrier dysfunction and cognitive impairment [39]. Two items representing hematopoietic and immune systems were also among the top-rated health deficits variables (Table 4) and were included in FI_r_: blood neutrophil count and red blood cell count (RBC). Neutrophils are the most abundant leukocyte in the periphery and are gaining increasing attention as a prognostic AD biomarker [40]. Neutrophils are hypothesized to contribute to AD progression through systemic inflammation and disturbance of the blood–brain barrier [41]. RBC is one of several red blood cell indices associated with AD and cognitive decline [42], and may reflect an array of pathological disturbances affecting brain function, i.e., B-vitamin deficiencies [43], anemia [44], and chronic kidney disease [45].

Cluster analysis revealed a higher degree of depressive symptomatology in the smaller, “frail” cluster 2, compared with cluster 1 (see Table 3). In turn, FI_r_ included both self-reported depressed mood, the NPI depression-item and GDS score as health deficits, reinforcing the strong link between frailty and depressive syndromes [46]. Depression is also a common manifestation in AD with prevalence estimates up to 50% [47], with associations with AD pathology including amyloid-β accumulation [48]. The present findings are intriguing as the ADNI study was designed to rule out subjects with clinical depression, and points to an important role even for subclinical symptoms. Indeed, one study found that even subthreshold symptoms of geriatric depression were related to AD-related neurodegeneration, which appeared to be independent of amyloid burden [49]. Self-reported low levels of energy and abnormal gait were also among the top variables that differed between the cluster subgroups. These characteristic frailty components might represent targets of prevention, as a randomized controlled clinical trial found that exercise was effective in reducing cognitive frailty [50]. Overall, the clustering approach identified a nuanced pattern of frailty-related health factors jointly contributing to the predictive ability of FI_r_.

### The relationship between frailty and future dementia risk varies across FI-variables

The degree of frailty predicted conversion from MCI to dementia across samples for the data-driven FI_r_ only. Although standard generated FIs (FI_s_, FI_c_) associated with future dementia risk in age, sex, and education-adjusted models, these results failed out-of-sample validation and the association did not remain when adjusting for baseline CDRSB scores. We are aware of few FI-studies of dementia risk with external validation, and most lack correction for baseline cognition using more comprehensive tools such as CDRSB. Many geriatric outpatient clinics have started implementing frailty assessment by means of FIs created using standard procedure. Given that validated tools for cognitive and functional assessment such as CDR exist, our findings question the addition of time-consuming assessment by means of standard FIs for clinical dementia workup unless their predictive abilities are improved following, e.g., a data-driven optimization procedure.

Several studies have employed prediction models of dementia due to AD [51], but to the best of our knowledge, this is the first study that does so based on subject clustering of health deficits related to frailty. Interestingly, only FI_r_, and not FI_s_ and FI_c_ were predictive of conversion to AD when including CDRSB as an additional covariate, suggesting that the clustering approach to selecting health deficits yields a frailty measure with added predictive value beyond global cognitive functioning. Based on the specific health differences between cluster subgroups, this finding is in line with the literature. For instance, differences in low energy and gait abnormalities fit well with the frailty construct, and studies indicating that physical activity associates with AD risk [e.g., 52].

### Limitations

The current findings should be interpreted with the following limitations taken into consideration. The selection of health variables was based on availability in the ADNI database, which was not originally designed to estimate frailty. Thus, our selection did not include certain phenotypical frailty measures such as weight loss, poor grip strength, or walking speed. The cut-points for the individual health deficits used to estimate FI are somewhat arbitrary, such as for blood pressure, and also applying data-driven approaches to different reference ranges might have improved the utility of the FI [53]. Another limitation is the lack of validation against traditional frailty endpoints, such as mortality, hospitalizations and falls. In exploratory analyses, we found that crude FI_r_ HR-estimates for mortality in ADNI were comparable to those in the literature [32]. Although FI_r_ was the only FI-variable correlating with mortality in fully adjusted analysis (Supplemental Table S5), superiority in the prediction of mortality and other frailty endpoints needs to be assessed in future research employing larger-scale databases. Furthermore, the dataset employed here did not allow for comparison of our data-driven revision of the accumulation of deficits model with results obtained by using the rival phenotype or physical frailty model [54].

To our knowledge, using FAMD-based subject clustering as an approach for selecting out features, such as health deficits is novel. In particular, selecting health deficits based on regression analyses of cluster belonging using FDR-adjusted p-value threshold has not been tested before. The sensitivity to differences in sample size might call for further development of our method using unbiased deficit selection for different population and sample sizes. One could even argue that a more appropriate approach would be to select features using a supervised approach [55]. However, an advantage to our unsupervised approach is that it more likely captures variance related to frailty per se compared to supervised methods, and is not bound to categorical definitions of diagnoses. Another advantage is that the present approach does not need information about the primary endpoint (e.g., mortality, incident dementia) for model training, importantly enabling FI development on novel datasets and clinical cohorts where prospective endpoint data are not yet available.

## Conclusion

Adding a data-driven supplemental step to the standard procedure for creating an FI improves prediction of cognitive status and future dementia risk. While the data-driven procedure employed here reduced the number of items included in an FI, the remaining selection adhered well with standard criteria outlined by Searle and colleagues [10] and included items reflecting core components of the frailty construct [1].

The two identified subject clusters from cluster analysis showed a unique constellation of health deficits which contributed to the stronger predictive ability of diagnosis and disease progression. In particular, our data-driven clustering analysis suggested a strong contribution of activities of daily living, polypharmacy, and tests reflecting immune, hematopoietic and cardiovascular systems, as well as several items of depressive symptomatology in AD risk stratification, even within a sample well-screened to rule out clinical depression. The results point to frailty––when measured using an FI with data-driven health deficit assessment––as a putative modifiable AD risk factor. The proposed data-driven procedure warrants further testing on other often-used frailty endpoints, such as mortality.

## Supplementary Information

Below is the link to the electronic supplementary material.

Supplementary file1 (DOCX 10523 KB)
