# Supplementary material for: Data-driven health deficit assessment improves a frailty index’s prediction of current cognitive status and future conversion to dementia: results from ADNI
Source: GeroScience. 2022 Oct 19;45(1):591–611. doi: 10.1007/s11357-022-00669-2 (PMC9886733; doi:10.1007/s11357-022-00669-2)
Supplement: Supplementary file 1 — Supplementary file1 (DOCX 10523 KB) [file 11357_2022_669_MOESM1_ESM.docx]

# Supplementary figures

**
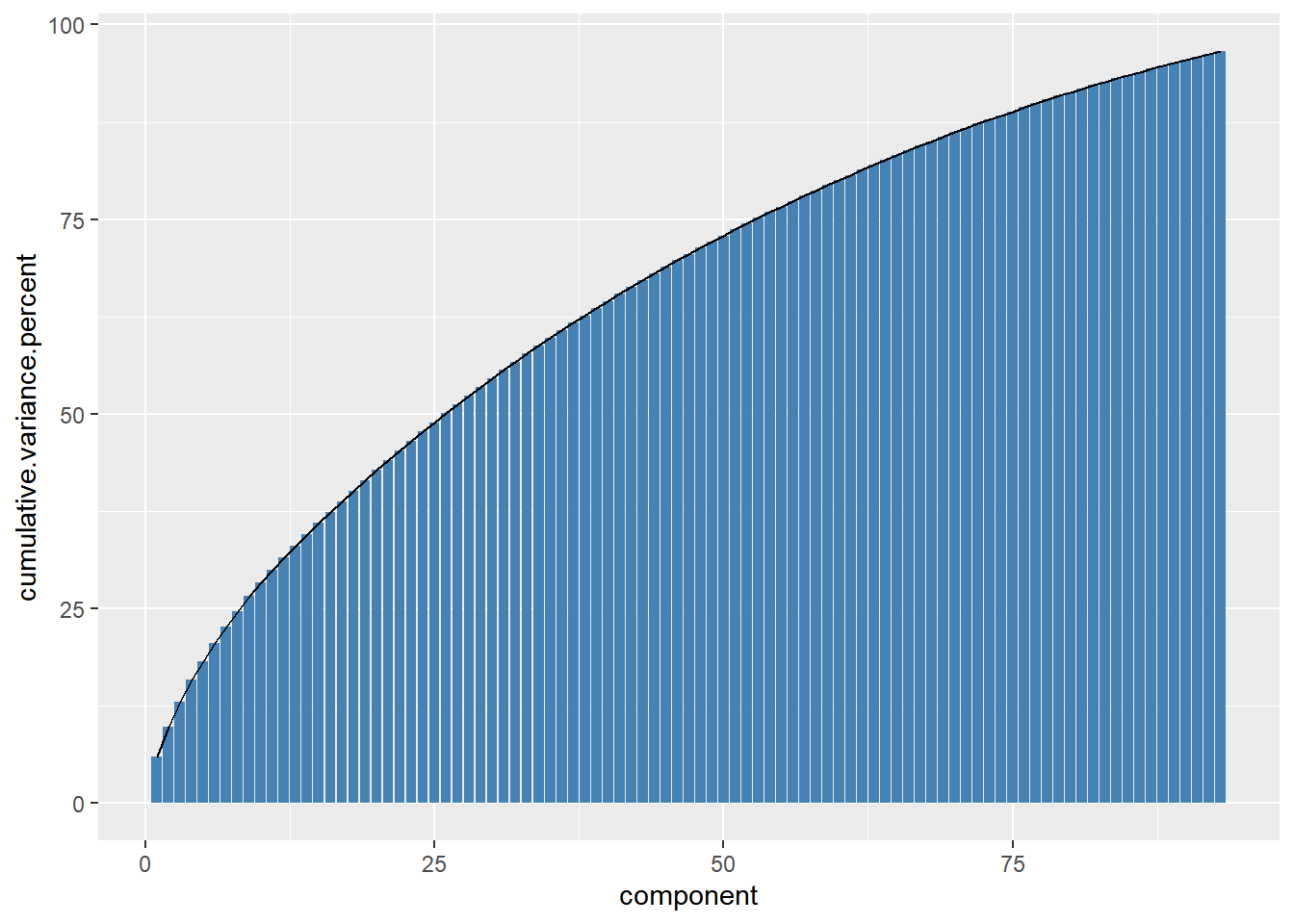
**


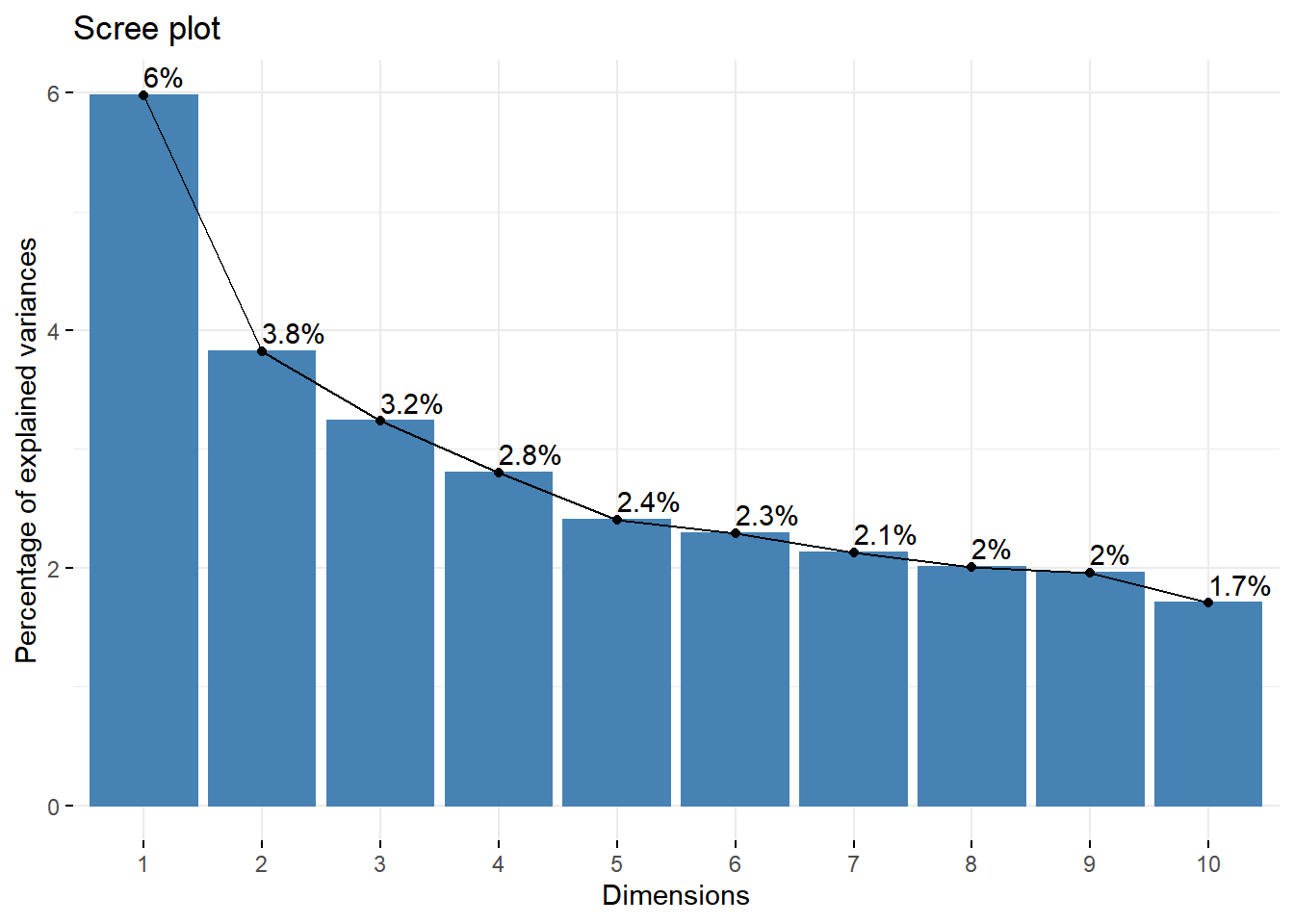


**Supplementary Fig. 1** Cumulative explained variance and scree plot of factor analysis of mixed data (FAMD) principal components. Top row shows the cumulative explained variance of 93 principal components (PCs) from factor analysis of mixed data (FAMD) on 93 health deficit variables selected according to a standard procedure. Bottom row shows percentage of explained variance from the first 10 PCs (i.e., "Dimensions")

#


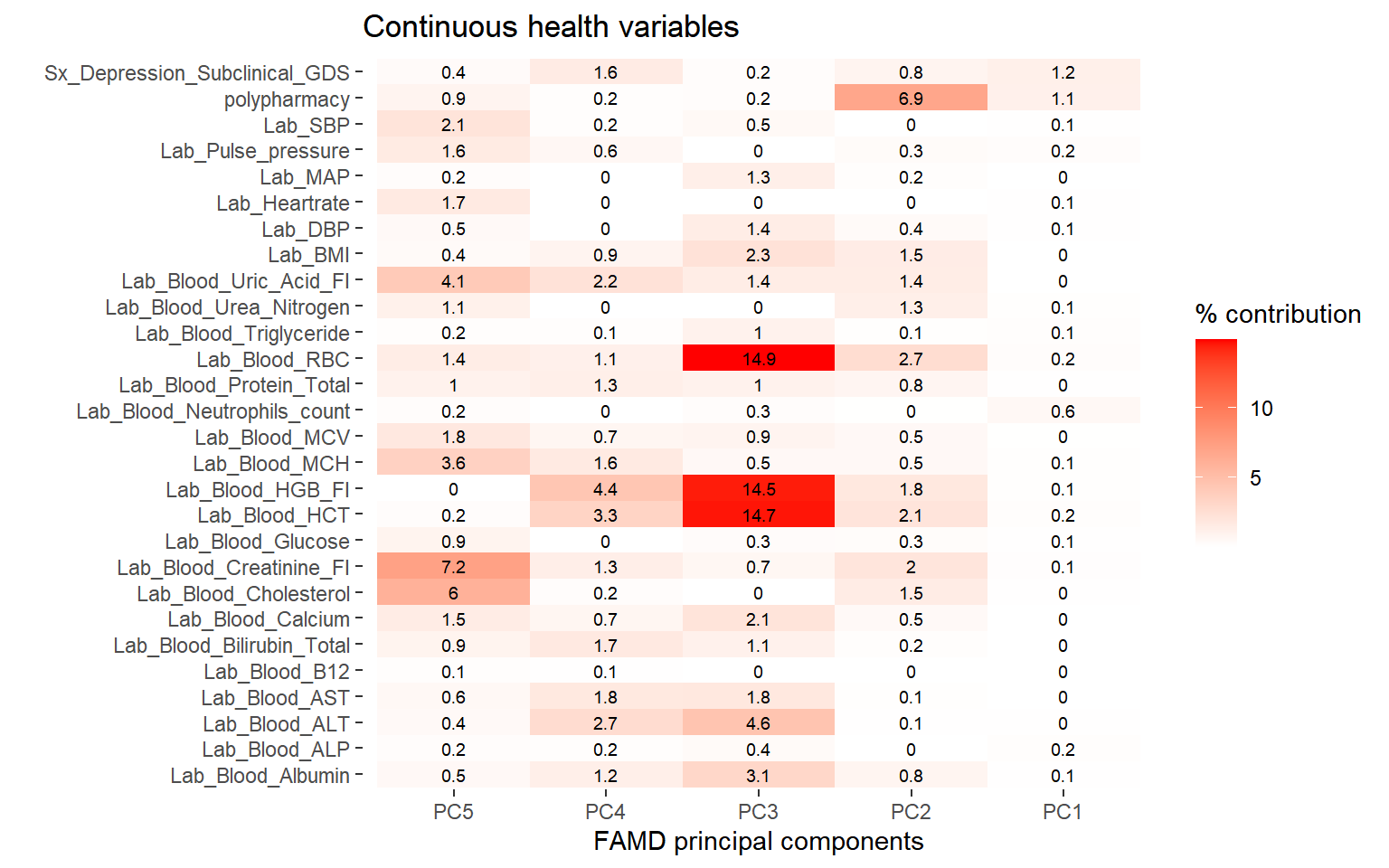
**Supplementary Fig. 2** Contribution of continuous health deficit variables to principal components 1 to 5. The figure shows the contribution of continuous health deficit variables to the first five principal components resulting from factor analysis of mixed data (FAMD) on 93 health deficit variables selected according to a standard procedure [1]


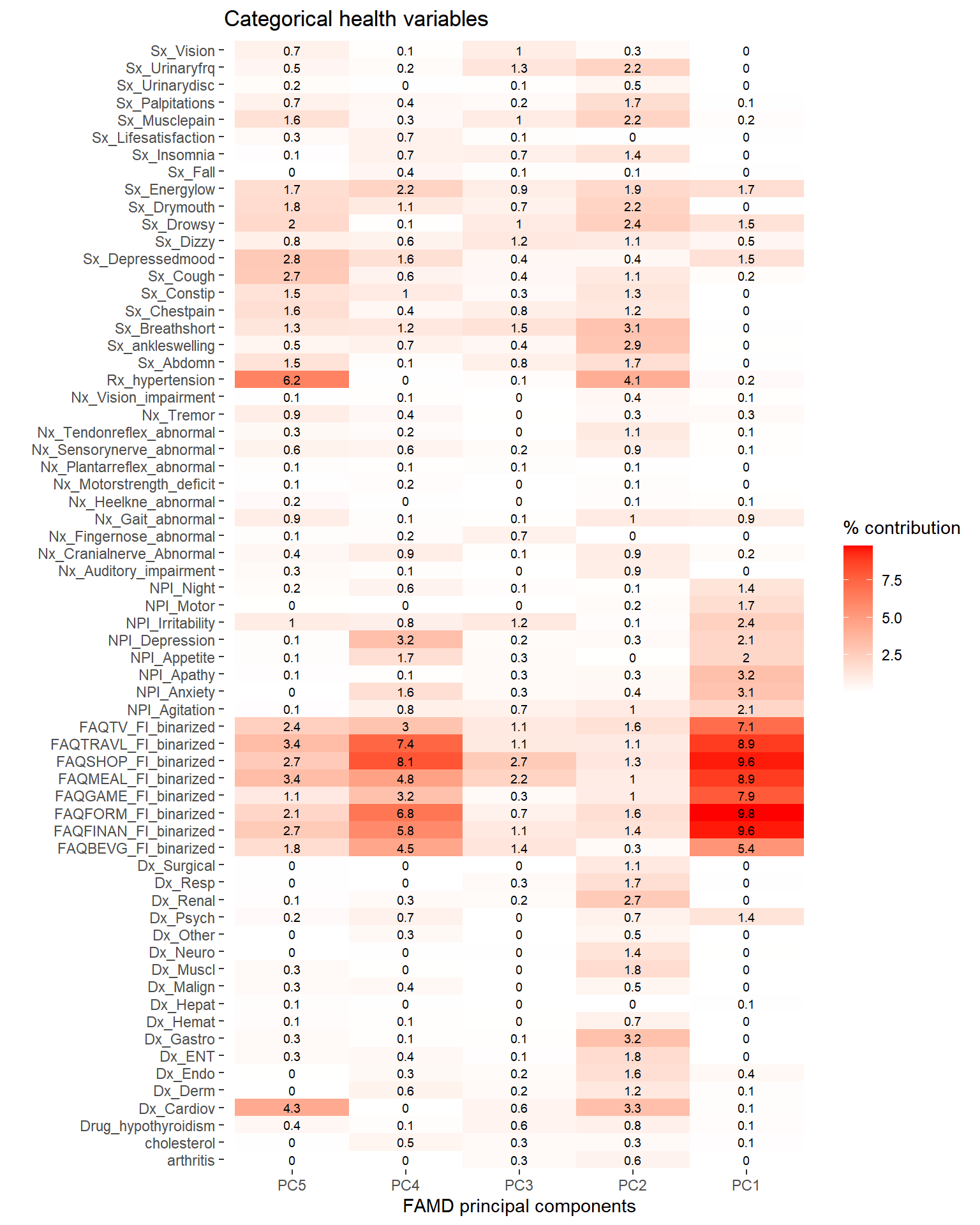


**Supplementary Fig. 3** Contribution of categorical health deficit variables to principal components 1 to 5 resulting from factor analysis of mixed data (FAMD) on 93 health deficit variables selected according to a standard procedure [1]

**Supplementary Fig. 4** Kaplan Meier curves illustrate survival probability during follow-up in sample tertiles for three different frailty indices (FI_s_, FI_r_, FI_c_) for development (ADNI1) and validation cohorts (ADNI2+GO). FI_s_ = A 93-item FI created according to standard procedure by the authors. FI_r_ = A 26-item FI created by adding a data-driven supplement to the standard procedure. FI_c_ = A 40-item FI created according to standard procedure [1] by Canevelli, et al., [2]

**Supplementary Fig. 5** Scatterplots with locally weighted smoothing lines illustrating the relationship between age and three different frailty indices (FI) in ADNI Development (ADNI1) and Validation samples (ADNI2+GO). *r*-values represent the Pearson correlation coefficient

**Supplementary Fig. 6** The figure shows area under the time-dependent ROC-curves for prediction of future dementia conversion in subjects with mild cognitive impairment at baseline for three frailty indices (FIs). Mean AUC = solid line, 95% confidence interval and bands = dotted lines, bands are the outermost. FI_s_ = A 93-item frailty index (FI) created according to standard procedure by the authors. FI_r_ = A 26-item FI created by adding a data-driven supplement to the standard procedure. FI_c_ = A 40-item FI created according to standard procedure by Canevelli, et al., [2]. Plots are shown for both development (ADNI1) and validation (ADNI2 and ADNI GO) samples

**Supplementary Fig. 7** The figure shows differences (mean = solid line, 95% confidence interval and bands = dotted lines, bands are the outermost) in AUC(t) between a data-driven frailty index (FI_r_) and two FIs generated according to standard procedure (FI_s_,FI_c_) for prediction of future dementia conversion in subjects with mild cognitive impairment at baseline. Plots are shown for development (ADNI1) and validation (ADNI2 and ADNI GO) samples and confirm greater AUC(t) for the data-driven FI_r_ over time compared with standard FIs

# Supplementary tables

**Supplementary Table 1: Categorical health deficit variables and thresholds used for dichotomization of deficits**

|  | **Coded as** |  |  |  |
| --- | --- | --- | --- | --- |
| **Categorical health deficit variables** | **0** | **0.25** | **0.5** | **1** |
| On medication for arthritis | No |  |  | Yes |
| On medication for elevated cholesterol | No |  |  | Yes |
| On medication for hypertension | No |  |  | Yes |
| On medication for hypothyroidism | No |  |  | Yes |
| History of cardiovascular disease | No |  |  | Yes |
| History of dermatologic-connective tissue disease | No |  |  | Yes |
| History of endocrine-metabolic disease | No |  |  | Yes |
| History of head, eyes, ears, nose, and throat diseases | No |  |  | Yes |
| History of gastrointestinal disease | No |  |  | Yes |
| History of hematopoietic-lymphatic disease | No |  |  | Yes |
| History of hepatic disease | No |  |  | Yes |
| History of malignancies | No |  |  | Yes |
| History of musculoskeletal disease | No |  |  | Yes |
| History of neurological (non-AD) disease | No |  |  | Yes |
| History of psychiatric disease | No |  |  | Yes |
| History of renal-genitourinary disease | No |  |  | Yes |
| History of respiratory disease | No |  |  | Yes |
| History of major surgical procedures | No |  |  | Yes |
| History of other diseases | No |  |  | Yes |
| FAQ, Writing checks, paying bills, or balancing checkbook | Independent /normal functioning (0) | Has difficulty, but does by self (1) | Requires assistance (2) | Dependent (3) |
| FAQ, Assembling tax records, business affairs, or other papers | Independent /normal functioning (0) | Has difficulty, but does by self (1) | Requires assistance (2) | Dependent (3) |
| FAQ, Heating water, making a cup of coffee | Independent /normal functioning (0) | Has difficulty, but does by self (1) | Requires assistance (2) | Dependent (3) |
| FAQ, Traveling out of the neighborhood) | Independent /normal functioning (0) | Has difficulty, but does by self (1) | Requires assistance (2) | Dependent (3) |
| FAQ, Preparing a balanced meal | Independent /normal functioning (0) | Has difficulty, but does by self (1) | Requires assistance (2) | Dependent (3) |
| FAQ, Paying attention to and understanding a TV program, book, or magazine | Independent /normal functioning (0) | Has difficulty, but does by self (1) | Requires assistance (2) | Dependent (3) |
| FAQ, Playing a game of skill such as bridge or chess | Independent /normal functioning (0) | Has difficulty, but does by self (1) | Requires assistance (2) | Dependent (3) |
| FAQ, Shopping alone for clothes, household | Independent /normal functioning (0) | Has difficulty, but does by self (1) | Requires assistance (2) | Dependent (3) |
| NPI, Agitation | No |  |  | Yes |
| NPI, Anxiety | No |  |  | Yes |
| NPI, Depression | No |  |  | Yes |
| NPI, Irritability | No |  |  | Yes |
| NPI, Sleep disturbance | No |  |  | Yes |
| NPI, Apathy | No |  |  | Yes |
| NPI, Aberrant motor behavior | No |  |  | Yes |
| NPI, Change in appetite or eating | No |  |  | Yes |
| Auditory impairment on physical exam | Absent |  |  | Present |
| Cranial nerve abnormality on physical exam | Normal |  |  | Abnormal |
| Finger to nose test | Normal |  |  | Abnormal |
| Heel-knee test | Normal |  |  | Abnormal |
| Gait on physical exam | Normal |  |  | Abnormal |
| Motor strength deficit on physical exam | Normal |  |  | Abnormal |
| Plantar reflex on physical exam | Normal |  |  | Abnormal |
| Tendon reflex test | Normal |  |  | Abnormal |
| Sensory nerve abnormality on physical exam | Normal |  |  | Abnormal |
| Tremor on physical exam | Absent |  |  | Present |
| Vision impairment on physical exam | Absent |  |  | Present |
| Symptoms from abdomen | Absent |  |  | Present |
| Self-reported ankle swelling | Absent |  |  | Present |
| Self-reported shortness of breath | Absent |  |  | Present |
| Self-reported chest pain | Absent |  |  | Present |
| Self-reported constipation | Absent |  |  | Present |
| Self-reported cough | Absent |  |  | Present |
| Self-reported depressed mood | Absent |  |  | Present |
| Self-reported dizziness | Absent |  |  | Present |
| Self-reported drowsiness | Absent |  |  | Present |
| Self-reported dry mouth | Absent |  |  | Present |
| Self-reported low energy | Absent |  |  | Present |
| Self-reported recent fall | Absent |  |  | Present |
| Self-reported insomnia | Absent |  |  | Present |
| Self-reported life satisfaction | Yes |  |  | No |
| Self-reported muscle pain | Absent |  |  | Present |
| Self-reported palpitations | Absent |  |  | Present |
| Self-reported urinary discomfort | Absent |  |  | Present |
| Self-reported urinary frequency | Absent |  |  | Present |
| Self-reported vision disturbance | Absent |  |  | Present |

Categorical Health deficit variables. FAQ = Functional Assessment Questionnaire. NPI = Neuropsychiatric Inventory.

**Supplementary Table 2: Quantitative health deficit variables and thresholds used for dichotomization of deficits**

| **Health variable (quantitative)** | **Lower threshold** | **Upper threshold** |
| --- | --- | --- |
| Blood Albumin (g/dL) | Male (age < 69) = 3.3  Male (age 69 to 80) = 3.3  Male (age > 80) = 3.0  Female (age < 69) = 3.3  Female (age 69 to 80) = 3.3  Female (age > 80) = 3.0 | Male (age < 69) = 4.9  Male (age 69 to 80) = 4.6  Male (age > 80) = 4.6  Female (age < 69) = 4.9  Female (age 69 to 80) = 4.6  Female (age > 80) = 4.6 |
| Blood Alkaline Phosphatase (ALP; U/L) | Any sex, any age = 35 | Male (age 50 to 60) = 131  Male (age 60 to 70) = 125  Male (age 70 to 80) = 130  Male (age > 80) = 125  Female (age 50 to 70) = 123  Female (age 70 to 90) = 135  Female (age > 90) = 140 |
| Blood Alanine Aminotransferase (ALT; U/L) | Any sex, any age = 6 | Male (age < 69) = 43  Male (age ≥ 69 = 35  Female (age < 69) = 34  Female (age ≥ 69) = 32 |
| Blood Aspartate aminotransferase (AST; U/L) | Male (any age) = 11  Female (any age) = 9 | Male (any age) = 36, Female (any age) = 34 |
| Blood Vitamin B12 (pg/mL) | Any sex, any age = 180 | Any sex, any age = 914 |
| Blood Total bilirubin (mg/dL) | Any sex, any age = 0.2 | Any sex, any age = 1.2 |
| Blood Calcium (mg/dL) | Any sex, any age = 8.3 | Any sex, any age = 10.6 |
| Blood Cholesterol (mg/dL) | Male (age < 60) = 170  Male (age 60 to 70) = 175  Male (age > 70) = 177  Female (age < 60) = 171  Female (age 60 to 70) = 188  Female (age > 70) = 207 | Male (age < 60) = 291  Male (age 60 to 70) = 298  Male (age > 70) = 300  Female (age < 60) = 291  Female (age 60 to 70) = 320  Female (age > 70) = 352 |
| Blood Creatinine (mg/dL) | Male (any age) = 0.5  Female (any age) = 0.4 | Male (age 50 to 70) = 1.3  Male (age 70 to 80) = 1.5  Male (age > 80) = 1.6  Female (age 50 to 70) = 1.1  Female (age 70 to 80) = 1.2  Female (age > 80) = 1.4 |
| Blood Glucose (mg/dL) | Any sex, any age = 70 | Any sex (age < 60) = 115  Any sex (age > 59) = 120 |
| Blood Hematocrit (%) | Male (age < 60) = 39  Male (age > 59) = 37  Femal (any age) = 34 | Male (age < 60) = 54  Male (age > 59) = 51  Femal (any age) = 48 |
| Blood Hemoglobin (g/dL) | Male (age < 60) = 12.7  Male (age > 59) = 12.5  Female (age < 60) = 11.6  Female (age > 59) = 11.5 | Male (age < 60) = 18.1  Male (age > 59) = 17.0  Female (age < 60) = 16.4  Female (age > 59) = 15.8 |
| Blood Mean corpuscular hemoglobin (MCH; pg) | Any sex, any age = 26 | Any sex, any age = 34 |
| Blood Mean corpuscular volume (MCV; fL) | Any sex (age < 60) = 79  Any sex (age > 59) = 80 | Male (age < 60) = 96  Female (age < 60) = 98  Any sex (age > 59) = 100 |
| Blood Neutrophil count (10^3^/μl) | Any sex, any age = 1.96 | Any sex, any age = 7.23 |
| Blood Total protein (g/dL) | Any sex (age < 60) = 6.1  Any sex (age > 59) = 6.0 | Any sex (age < 60) = 8.4  Any sex (age > 59) = 8.0 |
| Blood Red Blood Cell Count (RBC; 10^6^/μl) | Male (age < 60) = 4.5  Male (age > 59) = 4.0  Female (age < 60) = 4.1  Female (age > 59) = 3.9 | Male (age < 60) = 6.4  Male (age > 59) = 5.8  Female (age < 60) = 5.6  Female (age > 59) = 5.5 |
| Blood Triglycerides (mg/dL) | Male (any age) = 58  Female (age < 60) = 52  Female (age > 59) = 56 | Male (age < 60) = 320  Male (age > 59) = 260  Female (age < 60) = 262  Female (age > 59) = 240  Female (age < 60) =  Female (age > 59) = |
| Blood Urea nitrogen (BUN, mg/dL) | Any sex, any age = 4 | Any sex (age < 70) = 24  Any sex (age 70 to 80) = 29  Any sex (age > 80) = 34 |
| Blood Uric acid (mg/dL) | Any sex, any age = 2.5 | Male (any age) = 8.3 Female (any age) = 7.5 |
| Body mass index (BMI) | 18.5 | 30 |
| Diastolic blood pressure (mmHg) | 60 | 89 |
| Heart rate (count) | 60 | 99 |
| Mean arterial pressure (mmHg) | 70 | 105 |
| Pulse pressure (mmHg) | 30 | 65 |
| Systolic blood pressure (mmHg) | 90 | 139 |
| Number of medications (polypharmacy)* | No lower threshold | 5 |
| Elevated Geriatric Depression Scale (GDS) score** | No lower threshold | 0 |

Quantitative health deficit variables that were dichotomized (0 and 1). The dichotomized variable was coded as “0” if the health deficit variable value was within threshold values, and “1” if outside. For blood test results, we used the age- and gender-specific (if applicable) reference ranges reported by the ADNI laboratory. * Number self-reported medications excluding supplements: More than 5 medications were considered polypharmacy. **In ADNI, a GDS-score threshold of 6 was used as an exclusion-criterium to rule out participants with clinical depression; here, we considered scores from 1 to 6 as subclinical depression and scored these as a deficit if present.

**Supplementary Table 3: Binary classification in the development sample with class imbalance correction by undersampling**

| **Classification / Performance measure** | **FI-variable** | | |
| --- | --- | --- | --- |
| *HC vs. AD* | FI_s_ | FI_r_ | FI_c_ |
| Area under the curve | 0.75 (0.00) | 0.95 (0.00) | 0.81 (0.00) |
| Sensitivity | 0.71 (0.01) | 0.94 (0.00) | 0.75 (0.01) |
| Specificity | 0.67 (0.00) | 0.77 (0.00) | 0.70 (0.00) |
| PPV | 0.68 (0.00) | 0.81 (0.00) | 0.72 (0.00) |
| NPV | 0.70 (0.01) | 0.93 (0.00) | 0.74 (0.01) |
| F1-score | 0.69 (0.00) | 0.87 (0.00) | 0.73 (0.00) |
| *MCI vs. AD* | FI_s_ | FI_r_ | FI_c_ |
| Area under the curve | 0.68 (0.00) | 0.81 (0.00) | 0.72 (0.00) |
| Sensitivity | 0.71 (0.00) | 0.79 (0.00) | 0.72 (0.00) |
| Specificity | 0.62 (0.01) | 0.68 (0.00) | 0.64 (0.00) |
| PPV | 0.66 (0.01) | 0.72 (0.00) | 0.67 (0.01) |
| NPV | 0.69 (0.01) | 0.77 (0.01) | 0.70 (0.00) |
| F1-score | 0.68 (0.00) | 0.75 (0.00) | 0.69 (0.00) |
| *HC vs. MCI* | FI_s_ | FI_r_ | FI_c_ |
| Area under the curve | 0.59 (0.00) | 0.78 (0.00) | 0.61 (0.00) |
| Sensitivity | 0.60 (0.00) | 0.81 (0.00) | 0.63 (0.00) |
| Specificity | 0.54 (0.00) | 0.60 (0.00) | 0.52 (0.00) |
| PPV | 0.57 (0.00) | 0.67 (0.00) | 0.57 (0.00) |
| NPV | 0.58 (0.00) | 0.76 (0.00) | 0.59 (0.00) |
| F1-score | 0.58 (0.00) | 0.73 (0.00) | 0.60 (0.00) |

The binary classification analyses are corrected for class imbalance by using undersampling of the majority class. AD = Alzheimer’s disease dementia. FI_s_ = A 93-item frailty index (FI) created according to standard procedure by the authors. FI_r_ = A 26-item FI created by adding a data-driven supplement to the standard procedure. FI_c_ = A 40-item FI created according to standard procedure by Canevelli, et al., [2]. HC = Healthy cognitively normal control. MCI = Mild cognitive impairment. NPV = negative predictive value. PPV = positive predictive value. SD = Standard deviation.

**Supplementary table 4: Binary classification (HC versus AD) according to age and sex strata for three different FI-variables in the development sample**

| **FI-variable:** FI_s_ | **Age and sex stratum** | | | |
| --- | --- | --- | --- | --- |
| **Performance measure** | Young male | Old male | Young female | Old female |
| Area under the curve | 0.66 (0.02) | 0.79 (0.01) | 0.75 (0.02) | 0.78 (0.01) |
| Sensitivity | 0.97 (0.01) | 0.79 (0.01) | 0.83 (0.01) | 0.89 (0.01) |
| Specificity | 0.10 (0.02) | 0.71 (0.00) | 0.38 (0.02) | 0.42 (0.02) |
| PPV | 0.59 (0.01) | 0.75 (0.01) | 0.61 (0.01) | 0.67 (0.01) |
| NPV | 0.71 (0.02)^§^ | 0.78 (0.02) | 0.68 (0.05) | 0.79 (0.04) |
| F1-score | 0.73 (0.01) | 0.76 (0.01) | 0.70 (0.01) | 0.76 (0.01) |
| **FI-variable:** FI_r_ | **Age and sex stratum** | | | |
| **Performance measure** | Young male | Old male | Young female | Old female |
| Area under the curve | 0.95 (0.01) | 0.94 (0.01) | 0.96 (0.01) | 0.97 (0.01) |
| Sensitivity | 0.96 (0.00) | 0.93 (0.00) | 0.90 (0.00) | 0.98 (0.00) |
| Specificity | 0.67 (0.01) | 0.73 (0.01) | 0.78 (0.02) | 0.82 (0.02) |
| PPV | 0.81 (0.01) | 0.80 (0.01) | 0.85 (0.01) | 0.89 (0.01) |
| NPV | 0.95 (0.01) | 0.92 (0.01) | 0.89 (0.01) | 0.98 (0.01) |
| F1-score | 0.89 (0.00) | 0.85 (0.00) | 0.86 (0.01) | 0.93 (0.01) |
| **FI-variable:** FI_c_ | **Age and sex stratum** | | | |
| **Performance measure** | Young male | Old male | Young female | Old female |
| Area under the curve | 0.77 (0.02) | 0.80 (0.01) | 0.82 (0.02) | 0.85 (0.01) |
| Sensitivity | 0.90 (0.00) | 0.77 (0.01) | 0.84 (0.00) | 0.93 (0.00) |
| Specificity | 0.50 (0.02) | 0.66 (0.01) | 0.62 (0.02) | 0.63 (0.01) |
| PPV | 0.72 (0.01) | 0.71 (0.01) | 0.74 (0.01) | 0.78 (0.01) |
| NPV | 0.81 (0.03) | 0.75 (0.02) | 0.80 (0.03) | 0.90 (0.02) |
| F1-score | 0.79 (0.01) | 0.73 (0.01) | 0.78 (0.01) | 0.84 (0.00) |

AD = Alzheimer's disease dementia. HC = Healthy cognitively normal control. "Young" designates subjects < 75 years, "Old" >= 75 years. FI_s_ = A 93-item frailty index (FI) created according to standard procedure by the authors. FI_r_ = A 26-item FI created by adding a data-driven supplement to the standard procedure. FI_c_ = A 40-item FI created according to standard procedure by Canevelli, et al., [2]. NPV = negative predictive value. PPV = positive predictive value. SD = Standard deviation. ^§^Error margin is reported as standard error.

|  | **Sample** | | | |
| --- | --- | --- | --- | --- |
|  | Development (ADNI1) | | | |
| **FI-variable** | **Model 1^a^** | | **Model 2^b^** | |
|  | AHR (95% CI) | p-value | AHR (95% CI) | p-value |
| FI_s_ | 1.05 (1.02-1.09) | 0.002 | 1.03 (1.00-1.07) | 0.08 |
| FI_r_ | 1.04 (1.02-1.06) | <0.001 | 1.02 (1.01-1.04) | 0.01 |
| FI_c_ | 1.04 (1.02-1.06) | <0.001 | 1.02 (1.00-1.05) | 0.06 |
|  | Validation (ADNI2+GO) | | | |
| **FI-variable** | **Model 1^a^** | | **Model 2^b^** | |
|  | AHR (95% CI) | p-value | AHR (95% CI) | p-value |
| FI_s_ | 1.05 (1.01-1.09) | 0.008 | 1.03 (0.99-1.06) | 0.20 |
| FI_r_ | 1.03 (1.01-1.04) | 0.001 | 1.01 (0.99-1.03) | 0.25 |
| FI_c_ | 1.03 (1.01-1.06) | 0.01 | 1.01 (0.99-1.04) | 0.32 |

**Supplementary table 5: Association between FI-variables and mortality risk**

There were 86 deaths (11% of the total sample) during a median follow-up time of 1113 days in the development cohort, and 45 (6% of the total sample) during a median follow-up time of 1477 days in the validation cohort, respectively. Due to non-proportional hazards, we report average hazard ratios (AHRs) instead of standard HRs; AHRs were estimated using Prentice weights with censoring correction and robust variance estimation (see Methods). CI = confidence interval. FI_s_ = A 93-item frailty index (FI) created according to standard procedure by the authors. FI_r_ = A 26-item FI created by adding a data-driven supplement to the standard procedure. FI_c_ = A 40-item FI created according to standard procedure by Canevelli, et al., [2]. *All FI-variables were multiplied by 100 before entered into the models. ^a^Model 1 included age, sex and education as covariates. ^b^Model 2 included age, sex, education and Mini-mental state examination (MMSE) score.

## References

1. Searle SD, Mitnitski A, Gahbauer EA, Gill TM, Rockwood K: A standard procedure for creating a frailty index**.** *BMC Geriatrics* 2008, 8(1):24; <https://doi.org/10.1186/1471-2318-8-24>.

2. Canevelli M, Arisi I, Bacigalupo I, Arighi A, Galimberti D, Vanacore N, D’Onofrio M, Cesari M, Bruno G: Biomarkers and phenotypic expression in Alzheimer’s disease: exploring the contribution of frailty in the Alzheimer’s Disease Neuroimaging Initiative**.** *GeroScience* 2021, 43(2):1039-51; <https://doi.org/10.1007/s11357-020-00293-y>.
